# Differences in the relationship between pain and anxiety in total knee and hip arthroplasty: a longitudinal cross‐lagged analysis mediated by depression and pain catastrophizing

**DOI:** 10.1177/20494637241273905

**Published:** 2024-08-17

**Authors:** Ana Cristina Paredes, Patrício Costa, Márcia Costa, Patrícia Oliveira, Pedro Varanda, Armando Almeida, Patrícia R Pinto

**Affiliations:** 1 Life and Health Sciences Research Institute (ICVS), School of Medicine, 56059 University of Minho, Braga, Portugal; 2 ICVS/3B’s—PT Government Associate Laboratory, Braga/Guimarães, Portugal; 3 2CA-Braga, Clinical Academic Center, 389794 Hospital de Braga, Braga, Portugal; 4 Faculty of Psychology and Education Sciences, University of Porto, Porto, Portugal; 5 Orthopedics Department, Hospital of Braga, Braga, Portugal

**Keywords:** Acute postsurgical pain, anxiety, arthroplasty, pain catastrophizing

## Abstract

Acute postsurgical pain (APSP) is an important risk factor for pain chronification, with reports of being more intense after total knee arthroplasty (TKA) than after total hip arthroplasty (THA). Psychological variables have been associated with differences in postsurgical pain experience. This study aimed to analyse the longitudinal reciprocal association between pain and anxiety levels in patients undergoing TKA or THA, to investigate the moderator role of the type of surgery and to explore psychological mediators in the anxiety – pain association.

Patients undergoing TKA (*n* = 120) or THA (*n* = 109) were evaluated before surgery and in the acute postsurgical period (48 h postsurgery). Presurgical assessment comprised sociodemographic, pain-related and psychological variables (anxiety, depression, pain catastrophizing, self-efficacy, optimism and satisfaction with life). Postsurgical assessment focused on pain frequency, pain intensity and anxiety. Longitudinal associations were explored using cross-lagged panel models that included the indirect effect paths through possible mediators (pain catastrophizing and depression). Multigroup analyses compared TKA and THA.

In the global sample, higher APSP was predicted by higher presurgical pain and worse presurgical anxiety. Multigroup analyses revealed that worse APSP was predicted by higher presurgical anxiety in patients undergoing TKA and by higher presurgical pain in patients undergoing THA. Furthermore, there was a positive significant indirect effect of pain catastrophizing, but not depressive symptoms, in the relationship between presurgical anxiety and APSP in THA. Anxiety and APSP are differently interrelated in TKA and THA. Psychological characteristics could be managed before surgery to favour better APSP control and potentially prevent pain chronification after total joint arthroplasty.

## Introduction

Total joint arthroplasties are most commonly performed due to severe osteoarthritis, aiming to restore structural integrity and functionality of an affected joint.^1^ Acute postsurgical pain (APSP) is common after these interventions due to local injury and inflammation,^2^ being an expected and adaptive response. However, high levels of APSP are associated with increased suffering, perioperative complications, functional impairment, recovery delay and increased morbidity, making its effective control of paramount importance.^3–5^ Higher acute pain levels hinder active involvement in rehabilitation activities and may lead to poorer surgical outcomes.^6^ APSP has also been extensively associated with future pain chronification and is thus a relevant therapeutic target to prevent chronic postsurgical pain.^5^ Over the last decades, there have been considerable advances in understanding pain pathophysiology, developing modern analgesic techniques and publishing updated guidelines. However, effective acute pain control after surgery is still a challenge, with different surveys showing that a significant proportion of patients experience severe pain in the postoperative period.^7–9^ One plausible explanation for insufficient pain control is the variability in patient characteristics that may impact APSP,^10^ such as presurgical pain or psychological characteristics.^11,12^ Thus, a biopsychosocial perspective of pain is crucial to optimise APSP control,^13^ underlining the relevance of investigating factors that may influence it, namely, potentially modifiable characteristics such as psychological variables. In fact, variables such as anxiety, depression, pain catastrophizing and optimism can modulate (exacerbate or inhibit) pain perception^14,15^ and have been identified as relevant predictors of APSP after different types of surgery.^11,12,16^

In this scope, anxiety has been pointed as a relevant symptom to consider both before and after surgery. Indeed, clinical guidelines target anxiety as a potentially modifiable risk factor for worst surgical outcomes.^17,18^ Moreover, heightened levels of presurgical anxiety can progress to postsurgical anxiety.^19^ After surgery, anxiety is associated with APSP intensity, analgesia requirements, quality of recovery and wound healing^20–22^ Nonetheless, the potentially bidirectional relationship between these variables should be kept in mind. While anxiety may influence pain, it is also possible that higher pain intensity leads to increased anxiety.^23^

The relevance of psychological factors has also been shown specifically in orthopaedic surgeries, with pain catastrophizing, negative mood and optimism being recognised as predictors of APSP after total knee arthroplasty (TKA)^24–27^ and total hip arthroplasty (THA).^28^ Interestingly, one study even identified optimism as the most relevant predictor of APSP in a mixed TKA/THA sample, hinting at the relevance of positive psychological variables in this field.^29^ However, most studies of psychological predictors of acute post arthroplasty pain focus on TKA, with few reporting data separately for THA.^30,31^ This is a significant literature gap since there are well-documented differences in the outcomes of TKA and THA. During the acute postsurgical period, TKA patients experience more pain than THA patients,^32–34^ though the reasons for this are not well-understood.^34^ It is conceivable that psychological variables, along with other demographic, surgical and clinical factors, may help explain this variability.^30^

There is a need to further understand APSP after arthroplasty and to explore if conclusions derived from TKA can be generalised to other types of arthroplasties (e.g. THA). This study resorts to a cross-lagged panel model approach to describe the bidirectional relationships between variables at pre and postsurgical time points.^35^ Thus, the aims of this work were: (a) to analyse the longitudinal reciprocal association between pain and anxiety levels in patients undergoing TKA or THA, (b) to investigate if there is a moderator role of the type of surgery and (c) to explore psychological mediators in the anxiety – pain association.

## Methods

### Participants and procedure

Recruitment took place at the Orthopedics Unit of Hospital de Braga (Portugal), from June 2021 to December 2022. Patients were approached during a presurgical appointment with a specialist nurse and consecutively invited to participate if they met the inclusion criteria: (a) age ≥ 50 years old; (b) scheduled unilateral total joint arthroplasty due to osteoarthritis of the knee or hip; and (c) ability to understand written information and give informed consent. Exclusion criteria were: (a) revision surgery; (b) severe neurologic, psychiatric or organic diseases; and (c) contralateral knee or hip arthroplasty in the previous 6 months. In case of acceptance, the participants gave written informed consent before baseline assessment (T0, presurgical). Postsurgical assessment was conducted during the hospital stay, at 48 h postsurgery (T1).

Two hundred and 49 patients accepted to participate and were assessed before surgery. From the initial sample, six patients had their surgery postponed and 14 were excluded from the analyses (10 could not be assessed at 48 h, one had a subtrochanteric osteotomy and three had unicompartmental knee arthroplasty). Thus, the final sample included 229 participants.

All surgeries were performed by orthopaedic surgeons from the Orthopedics Unit of Hospital de Braga, who determined the appropriate surgical procedures for each patient. There were no research-related changes introduced to routine surgical or anaesthetic practices. Details concerning these procedures can be found in Supplemental Material 1.

Ethical approval was granted by the Ethics Committee from Hospital de Braga and Ethics Committee for Research in Life and Health Sciences from the University of Minho. All procedures followed international ethical guidelines for clinical studies involving humans.

### Measures

#### Presurgical assessment (T0)

- Sociodemographic and clinical questionnaire: sociodemographic information included data concerning age, sex, education, marital status and employment. The clinical section evaluated weight, height, comorbidities and pain characteristics (e.g. duration, other painful sites).
- American Society of Anesthesiologists physical status classification (ASA score)^36^: this information was retrieved from the clinical records to inform about pre-anaesthesia medical comorbidities. Each patient’s score is classified as ASA I (normal healthy patient), ASA II (patient with mild systemic disease), ASA III (severe systemic disease), ASA IV (life-threatening severe systemic disease), ASA V (moribund patient) or ASA VI (brain-dead patient).
- Brief Pain Inventory (BPI)^37^: evaluates pain intensity on an 11-point numerical rating scale (NRS; 0 = No pain; 10 = Worst pain imaginable). In this study, pre and postsurgical pain intensity at the affected joint were calculated by computing the mean score of pain ‘at its worst’ and ‘on the average’. At baseline, patients were asked about worst and average pain in the previous week. At 48h, the recall period was altered to encompass the acute period.
- Western Ontario and McMaster Osteoarthritis Index (WOMAC)^38^: disability was evaluated using a specific subscale from WOMAC (17 items). The subscale score is the sum of all items, with higher scores indicating more disability (range: 0-68).
- Coping Strategies Questionnaire-Revised (CSQ-R)^39^: pain catastrophizing was assessed with the corresponding six item subscale from the CSQ-R. The total score is the mean value of all answers (range: 1-5),^29^ with higher values indicating greater pain catastrophizing.
- Hospital Anxiety and Depression Scale (HADS)^40^: evaluates symptoms of general anxiety and depression in patients with medical illnesses.^41^ The questionnaire is comprised of two subscales (anxiety and depression) with seven items each, answered according to the previous week. When administered 48h after surgery, the anxiety items were answered considering the acute period. Each subscale scores range from 0 to 21, with higher scores representing higher symptomatology.
- Pain Self-Efficacy Questionnaire (PSEQ)^42^: evaluates confidence to engage in various activities of daily living, despite pain. The total score is the sum of the 10 items and varies from 0 to 60, with higher scores translating stronger self-efficacy beliefs.
- Life Orientation Test-Revised (LOT-R)^43^: derives an optimism score that is the sum of six items. The score may range from 0 to 24, with higher values translating more optimistic views.
- Satisfaction with Life Scale (SWLS)^44^: indicates the level of satisfaction with life through five items that derive a total score (range: 1-25). Higher scores indicate higher life satisfaction.

#### Postsurgical assessment (T1)

The assessment 48 h after surgery included pain frequency, pain intensity (BPI) and anxiety (HADS subscale). Pain frequency was assessed using the McGill Pain Questionnaire (MPQ),^45^ which groups pain as continuous (steady, constant), rhythmic (periodic, intermittent) or brief (momentary, transient). Additional surgical information was retrieved from medical records, namely, the type of anaesthesia, surgical approach, intra and postoperative analgesics.

### Statistical analyses

Participants’ characteristics are expressed as absolute and relative frequencies for categorical data (*n*, %) or mean and standard deviation for continuous variables (M ± SD). Only participants with complete data were included in the analyses (missing cases deleted listwise). The distribution properties of continuous variables were analysed through skewness (Sk) and kurtosis (Ku). Normality was assumed if Sk ± 2 and Ku ± 7^46^ and was guaranteed for all variables. Differences between patients undergoing TKA and THA were explored through independent samples *t*-tests or chi-square (*χ*^2^) tests, as appropriate. Paired samples *t*-tests analysed the pre to postsurgery differences in pain and anxiety levels. Effect size measures (Cohen’s d and Phy coefficient, ϕ) were computed to illustrate the meaningfulness of the results. The association of baseline (sociodemographic and psychological) variables with pain and anxiety levels was analysed through Pearson’s correlation coefficient. The analyses were performed using the IBM Statistical Package for the Social Sciences (SPSS), version 26 (Chicago, IL, USA). Statistical significance was set at *p* < .05.

**Figure 1.**
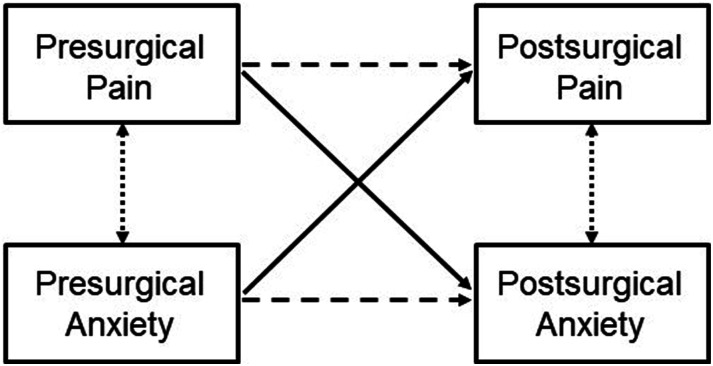
Illustration of the cross-lagged panel model used in this study. The solid lines represent the cross-lagged relationships between constructs and time points. Cross-sectional correlations are represented by dotted lines and autoregressive effects by dashed lines.

The longitudinal reciprocal relationship between anxiety levels and pain was explored through autoregressive cross-lagged panel models (CLPM). CLPM allows for simultaneous analysis of the multiple relationships specified in the models. A depiction of CLPM for this study can be seen in Figure 1. The model includes two constructs (anxiety and pain), assessed at two time points (before and after surgery), and the cross-lagged relationships between constructs and time points (solid lines in Figure 1). CLPM also controls for same-moment correlations (dotted lines) and autoregressive effects (stability over time, dashed lines).^35^ Age and sex were included as covariates in all models. The strength of the relationships was compared through standardised beta coefficients (β) and associated 95% confidence intervals (CI). Firstly, a CLPM was fit to the global study sample. Then, multigroup analysis was used to explore the moderator role of the type of surgery on the established relationships. Specific differences between groups were analysed through the associated critical ratios (CR). This calculation assumes a normal distribution of data, with a value of 1.96 indicating two-sided significance at the 5% level. When CR >1.96 for a given path, we can assume statistically significant differences.

**Figure 2.**
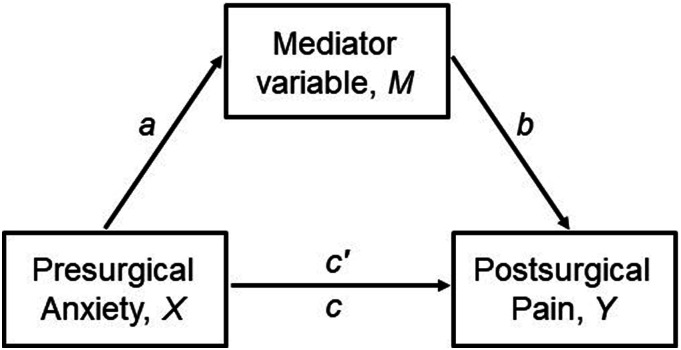
Illustration of a mediation model depicting the paths through which a mediator (M) influences the effect of the independent variable (X) on the outcome (Y). The arrows represent relationships between variables. Path c’ is the direct effect from X to Y, path axb is the indirect effect from X to Y, passing through M and path c accounts for both the direct and indirect effects.

Considering the association of psychological characteristics with anxiety and pain, we explored if they could mediate the association between presurgical anxiety and postsurgical pain that was revealed by the initial CLPM. Through mediation, it is possible to analyse if a given intervening variable (mediator, *M)* explains the mechanisms through which an independent variable (antecedent, *X*) causes an effect on a dependent variable (outcome, *Y*). A mediation model illustrates the different paths through which *X* can influence *Y*. This can happen through a *direct effect*, without intervention from *M* (path *c’*) or through an *indirect effect* from *X* to *Y,* passing through *M* (paths *a* x *b*).^47^ The combination of direct and indirect effects results in the total effects (path c). An illustration of mediation is shown in Figure 2. The indirect effect paths were added to the CLPM previously run without the mediator, for analysis purposes. The variables selected as potential mediators were those significantly associated with presurgical anxiety and postsurgical pain in previous correlation analyses (Pearson’s coefficient). Mediation tests were conducted based on 10 000 bootstrap samples to reduce sample variation.^47^ The results are reported for a 95% bias-corrected CI. Multigroup analyses by type of surgery were again used to investigate their role in the mediation results. CLPM and mediation models were fitted using maximum likelihood estimation in IBM SPSS AMOS, version 25.

The overall goodness-of-fit for the models was evaluated considering the following indices and thresholds: chi-square (*p* > .05), root mean square error of approximation (RMSEA <0.06) and comparative fit index (CFI >0.95).^48^ Sample size is within the recommended 10:1 ratio of cases to parameters.^49^ For the CLPM with indirect effect paths (20 parameters), 200 participants would be required. Thus, the study was well-powered to detect statistically significant effects.

To evaluate the longitudinal invariance of the anxiety assessment (HADS subscale), we used IBM SPSS AMOS v. 25 to specify two latent variables that correspond to anxiety assessment at T0 (baseline) and T1 (acute postsurgical period) (Supplemental Material 2). The model was estimated without any constraints to test if the factor structure is similar between time points (configural invariance). The analysis of the model’s fit indices, particularly CFI and RMSEA, shows that anxiety assessment has adequate longitudinal measurement invariance (χ^2^ (134) = 221.4, *p* < .001, CFI = 0.912, RMSEA = 0.054).

## Results

### Comparison of pre and postsurgical characteristics of TKA and THA patients

This study included 229 participants, of which 120 (52.4%) had TKA and 109 (47.6%) had THA. Baseline patient characteristics, postsurgical outcomes and comparison between TKA and THA are described in Table 1. Information regarding sample race and socioeconomic status were not collected. Mean age was 66.8 years old (SD = 7.6), with about half of the participants being female (123, 53.7%). The mean duration of presurgical pain was 79 months (approximately six and a half years, SD = 80.3 months). ASA score included 5 (2.2%) cases of ASA I, 165 (72.1%) ASA II and 59 (25.8%) ASA III.

**Table 1.** Description of pre and postsurgical characteristics and between-group differences.

Variable	Global sample (*n* = 229)	TKA (*n* = 120)	THA (*n* = 109)	Test statistics		
				*t*/χ^2^ (df)	*p*	d/ϕ
Presurgical (baseline, T0)						
Sex (female)	123 (53.7%)	85 (70.8%)	38 (34.9%)	29.726 (1)	**<0.001**	0.22
Age	66.76 ± 7.60	68.58 ± 6.98	64.75 ± 7.79	3.918 (227)	**<0.001**	0.52
Marital status (married)	179 (78.2%)	93 (77.5%)	86 (78.9%)	0.066 (1)	0.798	0.02
Education (≤ 4^th^ grade)	148 (64.6%)	88 (73.3%)	60 (55.0%)	8.356 (1)	**0.004**	0.19
Occupation (employed)	51 (22.3%)	20 (16.7%)	31 (28.4%)	4.574 (1)	**0.032**	0.14
BMI	29.29 ± 4.54	29.93 ± 4.32	28.59 ± 4.69	2.240 (227)	**0.026**	0.30
Nr. comorbidities	2.77 ± 167	2.87 ± 1.46	2.66 ± 1.88	0.922 (203)	0.358	0.12
Pain duration (months)	78.98 ± 80.29	91.48 ± 73.85	65.22 ± 85.06	2.500 (227)	**0.013**	0.33
Other painful sites (yes)	142 (62%)	84 (70.0%)	58 (53.2%)	6.834 (1)	**0.009**	0.17
Presurgical pain intensity (NRS)	6.63 ± 1.36	6.66 ± 1.38	6.61 ± 1.34	0.293 (227)	0.770	0.04
Disability (WOMAC)	29.31 ± 9.89	26.91 ± 8.25	31.96 ± 10.86	-3.938 (201)	**<0.001**	0.57
Anxiety (HADS)	5.42 ± 4.51	5.91 ± 4.48	4.88 ± 4.51	1.729 (227)	0.085	0.23
Depression (HADS)	3.21 ± 3.05	2.96 ± 2.72	3.48 ± 3.37	-1.275 (207)	0.204	0.18
Pain catastrophizing (CSQ-R)	1.90 ± 0.96	1.85 ± 0.90	1.95 ± 1.02	-0.750 (227)	0.454	0.10
Self-efficacy (PSEQ)	39.97 ± 10.57	41.57 ± 9.14	38.20 ± 11.73	2.404 (204)	**0.017**	0.34
Optimism (LOT-R)	16.44 ± 4.97	16.05 ± 4.88	16.86 ± 5.04	-1.236 (227)	0.217	0.16
Satisfaction with life (SWLS)	18.16 ± 4.318	18.28 ± 4.16	18.02 ± 4.51	0.463 (227)	0.644	0.06
Postsurgical (48h, T1)						
Surgery side (right)	117 (51.1%)	62 (51.7%)	55 (50.5%)	0.033 (1)	0.855	0.01
Length of stay (days)	4.77 (3.76%)	5.32 ± 4.00	4.17 ± 3.41	2.316 (227)	**0.021**	0.31
Pain frequency (MPQ)				14.502 (2)	**0.001**	0.25
Continuous	62 (27.1%)	43 (35.8%)	19 (17.4%)			
Rhythmic	74 (32.3%)	41 (34.2%)	33 (30.3%)			
Brief	91 (39.7%)	35 (29.2%)	56 (51.4%)			
Postsurgical pain intensity (NRS)	5.68 ± 2.06	6.12 ± 2.00	5.20 ± 2.03	3.451 (227)	**0.001**	0.46
Postsurgical anxiety (HADS)	2.17 ± 2.79	2.30 ± 2.83	2.02 ± 2.75	0.762 (227)	0.447	0.10

Note: Bold values are statistically significant at *p* < .05.

Abbreviations: TKA, total knee arthroplasty; THA, total hip arthroplasty; BMI, body mass index; NRS, numerical rating scale; VAS, visual analogue scale; HADS, Hospital Anxiety and Depression Scale; CSQ-R, Coping Strategies Questionnaire – Revised; PSEQ, Pain Self-Efficacy Questionnaire; LOT-R, Life Orientation Test – Revised; SWLS, Satisfaction with Life Scale; MPQ, McGill Pain Questionnaire.

There were statistically significant differences between patients undergoing TKA and THA in terms of sex (χ^2^ (1) = 29.7, *p* < .001), age (*t* (227) = 3.9, *p* < .001), education (χ^2^ (1) = 8.4, *p* = .004), occupation (χ^2^ (1) = 4.6, *p* = .032), body mass index (BMI) (*t* (227) = 2.2, *p* = .026), presurgical pain duration (*t* (227) = 2.5, *p* = .013), presence of other painful sites (χ^2^ (1) = 6.8, *p* = .009), disability (*t* (201) = -3.9, *p* < .001), self-efficacy (*t* (204) = 2.4, *p* = .017), length of stay (*t* (227) = 2.3, *p* = .021), postsurgical pain frequency (χ^2^ (2) = 14.5, *p* = .001) and intensity (*t* (227) = 3.5, *p* = .001. More women than men underwent knee surgery. TKA patients were older, with lower education levels, less often employed and had higher BMI. More patients with gonarthrosis than coxarthrosis reported other painful sites, they had longer pain duration, lower disability and higher pain self-efficacy levels. Patients who had TKA stayed more days at the hospital on average than THA, reported more continuous pain and had higher acute pain intensity. Paired samples t-tests (not shown) confirmed that pain and anxiety levels were significantly higher at baseline than at 48h (pain intensity: *t* (228) = 6.5, *p* < .011; anxiety: *t* (228) = 11.2, *p* < .001).

### Relationship of sex, age and baseline psychological characteristics with pre and postsurgical pain and anxiety levels

The association between study variables is detailed in Table 2. Female and younger patients reported higher pain (sex: r_pb_ = 0.17, *p* = .012; age: r = −0.15, *p* = .024) and anxiety (sex: r_pb_ = 0.25, *p* < .001; age: r = −0.18, *p* = .005) before surgery, but not in the acute postsurgical period. Patients with more intense pain before surgery had higher anxiety levels (r = 0.256, *p* < .001), depressive symptoms (r = 0.233, *p* < .001) and pain catastrophizing (r = 0.257, *p* < .001) and lower pain self-efficacy (r = −0.271, *p* < .001) and satisfaction with life (r = −0.197, *p* = .003). After surgery, APSP was positively associated with depressive symptoms (r = 0.14, *p* = .037) and pain catastrophizing (r = 0.16, *p* = .013).

**Table 2.** Relationship of baseline variables with pre and postsurgical pain and anxiety levels (global sample).

	Pain		Anxiety	
	Baseline	48h	Baseline	48h
Presurgical (baseline, T0)				
Sex	0.165*	0.113	0.250***	0.109
Age	−0.149*	−0.060	−0.184**	0.023
Pain	-	0.207**	0.256***	0.016
Anxiety (HADS)	0.256***	0.193**	-	0.350***
Depression (HADS)	0.233***	0.138*	0.627***	0.322***
Pain catastrophizing (CSQ-R)	0.257***	0.163*	0.545***	0.222***
Self-efficacy (PSEQ)	−0.271***	−0.081	−0.347***	−0.186**
Optimism (LOT-R)	−0.092	−0.022	−0.457***	−0.291***
Satisfaction with life (SWLS)	−0.197**	0.014	−0.438***	−0.239***
Postsurgical (48h, T1)				
Pain	0.207**	-	0.193**	0.286***
Anxiety (HADS)	0.016	0.286***	0.350***	-

Note: Bold values are statistically significant at *p* < .05 (*p < .05, **p < .01, ***p < .001).

Abbreviations: HADS, Hospital Anxiety and Depression Scale; CSQ-R, Coping Strategies Questionnaire – Revised; PSEQ, Pain Self-efficacy Questionnaire; LOT-R, Life Orientation Test – Revised; SWLS, Satisfaction with Life Scale; MPQ, McGill Pain Questionnaire.

All baseline psychological variables were significantly associated with presurgical anxiety levels (depression: r = 0.627, *p* < .001; pain catastrophizing: r = 0.545, *p* < .001; pain self-efficacy: r = −0.374, *p* < .001; optimism: r = −0.457, *p* < .001 and satisfaction with life: r = −0.438, *p* < .001) and with postsurgical anxiety levels (depression: r = 0.322, *p* < .001; pain catastrophizing: r = 0.222, *p* < .001, pain self-efficacy: r = −0.186, *p* = .005; optimism: r = −0.291, *p* < .001 and satisfaction with life: r = −0.239, *p* < .001). Higher depression symptomatology and pain catastrophizing, and lower pain self-efficacy, optimism and satisfaction with life were associated with more anxiety symptomatology at both time points.

### Longitudinal reciprocal relationships between anxiety and pain

Table 3 details CLPM results for the global sample and multigroup comparisons. The models showed an overall good fit (global sample: χ^2^ (4) = 2.6, *p* = .623; RMSEA = 0; CFI = 1; multigroup: χ^2^ (8) = 11.5, *p* = .175; RMSEA = 0.044; CFI = 0.969) (Table 4).

**Table 3.** Standardised estimates for CLPM of relationships between anxiety levels and surgical pain, and multigroup comparisons.

	Global sample			TKA			THA			Critical ratio (Z)*
Path	β	95% CI	*p*	β	95% CI	*p*	β	95% CI	*p*	
1: Pain T0 → Pain T1	0.169	[0.025, 0.323]	**0.011**	0.127	[-0.081, 0.328]	0.159	0.238	[0.014, 0.444]	**0.015**	0.893
2: Pain T0 → Anxiety T1	−0.079	[-0.199, 0.059]	0.216	−0.104	[-0.262, 0.073]	0.246	−0.053	[-0.248, 0.153]	0.557	0.398
3: Anxiety T0 → Pain T1	0.150	[0.002, 0.291]	**0.024**	0.226	[0.040, 0.400]	**0.012**	0.015	[-0.215, 0.246]	0.879	−1.582
4: Anxiety T0 → Anxiety T1	0.371	[0.247, 0.491]	**<0.001**	0.299	[0.130, 0.457]	**<0.001**	0.444	[0.257, 0.607]	**<0.001**	1.022

Note: Bold values are statistically significant at *p* < .05.

* Critical ratio (CR) calculation assumes a normal distribution of data, with a value of 1.96 indicating two-sided significance at the 5% level. When CR >1.96 for a given path, statistically significant differences can be assumed.

Abbreviations: CLPM, cross-lagged panel model; TKA, total knee arthroplasty; THA, total hip arthroplasty; CI, confidence interval.

**Table 4.** Model fit statistics for the CLPM of relationships between anxiety levels and surgical pain.

Model	χ^2^ (df)	*p*	RMSEA [90% CI]	CFI
Global sample				
No mediator	2.622 (4)	0.623	0 [0 – 0.082]	1
Mediator: Depression	10.734 (8)	0.217	0.039 [0 – 0.092]	0.988
Mediator: Pain catastrophizing	9.277 (8)	0.319	0.026 [0 – 0.085]	0.993
Multigroup				
No mediator	11.490 (8)	0.175	0.044 (0 – 0.096)	0.969
Mediator: Depression	23.306 (16)	0.106	0.045 [0 – 0.082]	0.969
Mediator: Pain catastrophizing	19.986 (16)	0.221	0.033 [0 – 0.033]	0.980

Abbreviations: CLPM, cross-lagged panel model; RMSEA, root mean square error of approximation; CI, confidence interval; CFI, comparative fit index.

**Figure 3.**
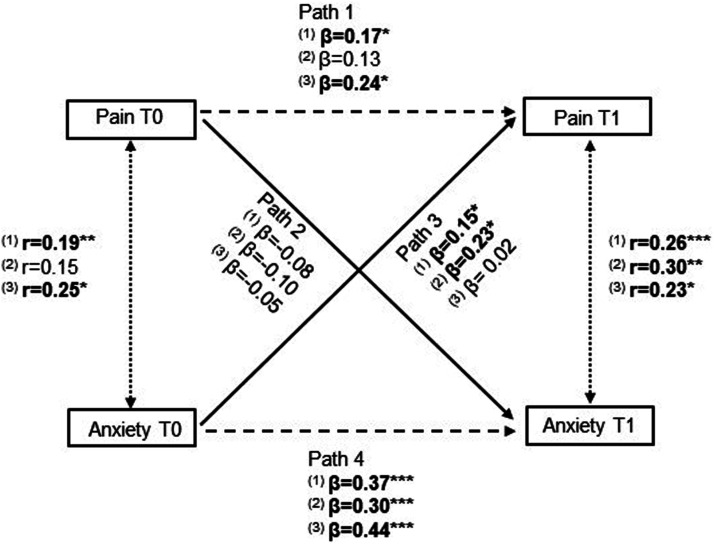
Illustration of the cross-lagged panel model between anxiety and pain for the global sample (1), TKA (2) and THA (3). The solid lines represent the cross-lagged relationships between constructs and time points. Cross-sectional correlations are represented by dotted lines and autoregressive effects by dashed lines. For simplicity, the included covariates and error terms are not depicted. Bold values are statistically significant at *p* < .05 (***p < .001, **p < .01, *p < .05).

The CLPM is depicted in Figure 3. For the global sample, there were statistically significant associations in three of the analysed paths (paths 1, 3 and 4). Baseline pain significantly predicted 48h pain (path 1: β = 0.17, *p* = .011) but was not associated with anxiety levels at 48h (path 2: β = −0.08, *p* = .216). On the other hand, baseline anxiety intensity predicted both 48h pain (path 3: β = 0.15, *p* = .024) and 48h anxiety levels (path 4: β = 0.37, *p* < .001). In the multigroup analyses, baseline pain continued to show a non-significant association with 48h anxiety levels in both surgeries (path 2, TKA: β = −0.10, *p* = .246; THA: β = −0.05, *p* = .557), while baseline anxiety predicted 48h anxiety intensity in both surgeries (path 4, TKA: β = 0.37, *p* < .001; THA: β = 0.44, *p* < .001). Path 1 (baseline pain predicting 48h pain) was only statistically significant in THA (β = 0.24, *p* = .015) and path 3 (baseline anxiety predicting 48h pain) was only statistically significant in TKA patients (β = 0.23, *p* = .012). That is, postsurgical pain was predicted by presurgical pain in patients undergoing THA and by presurgical anxiety levels in patients undergoing TKA. However, the critical differences ratios did not identify statistically significant differences in these paths, between the two groups (all *p* > .05; |Z values|<1.96). The full CLPM with standard estimates can be found in Supplemental Material 3.

### Mediation analyses

**Figure 4.**
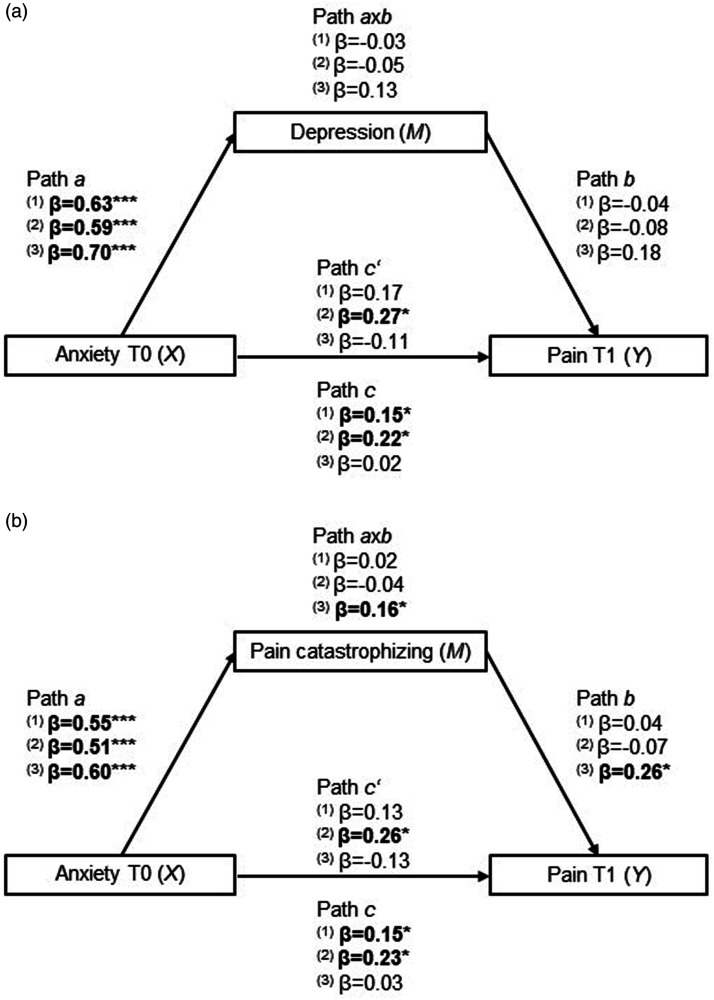
Illustration of the mediation analyses for the global sample (1), TKA (2) and THA (3). Depression (a) and pain catastrophizing (b) were tested as mediators (M) in the association between presurgical anxiety (X) and postsurgical pain (Y). The arrows represent relationships between variables. Path c’ is the direct effect from X to Y, path axb is the indirect effect from X to Y, passing through M and path c accounts for both the direct and indirect effects. Bold values are statistically significant at *p* < .05 (***p < .001, *p < .05).

Depressive symptoms and pain catastrophizing were tested as possible mediators in the relationship between presurgical anxiety levels and postsurgical pain, as they were significantly associated with both constructs (Table 2). Table 5 and Figure 4(a) and (b) show the results for the models including the potential mediating variables. All models testing mediation within CLPM showed adequate goodness-of-fit measures (Table 4). The full structural models and associated standard estimates can be found in Supplemental Material 4.

**Table 5.** Standardised estimates for CLPM including potential mediators in the relationship between presurgical anxiety levels (T0) and postsurgical pain (T1).

Model	Total effects (*c*)			Direct effects (*c’*)			Indirect effects (*a* x *b*)		
	β	95% CI	*p*	β	95% CI	*p*	β	95% CI	*p*
Mediator: Depression									
Global sample	0.149	[0.000, 0.136]	**0.048**	0.174	[-0.006, 0.340]	0.060	−0.025	[-0.122, 0.066]	0.544
TKA	0.224	[0.040, 0.397]	**0.015**	0.272	[0.045, 0.471]	**0.022**	−0.048	[-0.151, 0.056]	0.330
THA	0.018	[-0.214, 0.250]	0.890	−0.111	[-0.453, 0.185]	0.478	0.129	[-0.024, 0.340]	0.095
Mediator: Pain catastrophizing									
Global sample	0.151	[0.004, 0.293]	**0.043**	0.130	[-0.065, 0.313]	0.194	0.022	[-0.070, 0.116]	0.642
TKA	0.225	[0.039, 0.398]	**0.015**	0.262	[0.037, 0.468]	**0.022**	−0.037	[-0.153, 0.076]	0.483
THA	0.029	[-0.201, 0.259]	0.812	−0.128	[-0.389, 0.164]	0.387	0.157	[0.027, 0.306]	**0.019**

Note: Bold values are statistically significant at *p* < .05.

Abbreviations: CLPM, cross-lagged panel model; TKA, total knee arthroplasty; THA, total hip arthroplasty; CI, confidence interval.

When the variable ‘depressive symptoms’ was included to be tested as a mediator (Figure 4(a)), the results revealed statistically significant total effects for the global sample (β = 0.15, *p* = .048) and TKA (β = 0.22, *p* = .015) and significant direct effects for TKA only (β = 0.27, *p* = .022). In the THA group, there were no significant effects. Depression symptomatology was not a significant mediator in any of the tested models.

When analysing pain catastrophizing as a mediator (Figure 4(b)), there was a statistically significant total effect (β = 0.15, *p* = .043), but no mediation (indirect effects: β = 0.02, *p* = .642), in the global sample. In TKA, there were significant total (β = 0.23, *p* = .015) and direct effects (β = 0.26, *p* = .022) but no indirect effects (β = −0.04, *p* = .483). In THA, pain catastrophizing mediated the effect from baseline anxiety levels to 48 h pain (indirect effect: β = 0.16, *p* = .019).

## Discussion

This study analysed the longitudinal reciprocal associations between pre/postsurgical pain and pre/postsurgical anxiety levels in patients undergoing total knee or hip arthroplasty (TKA/THA) and explored psychological mediators of these relationships. The type of surgery was analysed as a moderator. Results showed that, for the global sample, higher acute postsurgical pain (APSP) was predicted by higher presurgical pain and higher presurgical anxiety symptomatology. However, postsurgical anxiety was only predicted by presurgical anxiety. These associations were different according to the type of surgery, with APSP being predicted only by presurgical anxiety in TKA and only by presurgical pain in THA. However, the critical ratios did not show significant differences between the two groups. Notwithstanding, mediation analysis showed that, specifically in THA patients, presurgical anxiety levels influenced APSP through pain catastrophizing.

In the present study, participants with knee osteoarthritis more frequently reported having other painful sites and had longer pain duration at the time of surgery. Indeed, there is generally a shorter time between symptom onset and surgery for THA than for TKA.^50^ This may be due to a more restricted joint range of motion typically experienced by patients with hip osteoarthritis that imposes more limitations.^51^ This assumption matches current results, with THA patients having shorter pain duration, but higher disability, which probably also underlies the differences in presurgical self-efficacy. Also of note is the fact that most patients undergoing TKA were women. Since women tend to undergo surgery at more advanced arthritis stages than men, it is possible that sex differences also account for these findings.^52,53^

Other studies have compared psychosocial characteristics before TKA and THA and concluded that most variables did not differ. There were some reported differences in optimism,^33^ emotional well-being^54^ or quality-of-life,^55^ but the findings were inconclusive in terms of which surgical group had better psychosocial status.

After surgery, APSP was higher in TKA patients, as expected.^34^ It is not clear why TKA causes higher APSP, especially because THA requires more tissue damage to expose the joint than TKA.^56^ Thus, identifying clinical and psychological determinants of APSP remains a relevant research field. For APSP control, the majority of TKA patients had a continuous disposable infusion balloon, while THA patients mostly received intravenous analgesia. It could be suggested that these differences influenced differences in APSP, but similar conclusions have been reached even when anaesthetic and analgesic strategies were uniform for TKA and THA.^32^ Thus, it is unlikely that the differences in this study are better explained by different anaesthetic and analgesic procedures.

The results showed a cross-sectional association between presurgical pain and psychological variables, both negative (anxiety, depression and pain catastrophizing) and positive (self-efficacy and satisfaction with life). This is consistent with the literature, which shows that osteoarthritis patients with higher anxiety, depression or pain catastrophizing likely report more pain.^57–59^ Conversely, positive emotions and expectations are associated with less intense pain.^59–61^ Consistent results were found at 48h, showing a same-moment significant positive association between pain intensity and anxiety levels. It is not possible to derive causal inferences from these results, but they highlight the interrelationship between pain and emotional well-being and the relevance of a biopsychosocial understanding of patients undergoing arthroplasty.^62^ A longitudinal correlation was demonstrated between presurgical negative psychological variables and APSP, which was not evident for positive psychological variables. Interestingly, optimism has emerged as the most important predictor of acute pain after TKA/THA,^29^ but it was not correlated with either pre or postsurgical pain intensity in this sample. In fact, optimism is frequently associated with pain,^16,63^ but there are also inconclusive reports of its effect on pain intensity after TKA and THA.^64^

Globally, the present findings are consistent with literature on psychological predictors of APSP, which pinpoints anxiety, depression and pain catastrophizing as risk factors for more intense pain.^11,12,16^ Anxiety is the psychological variable most frequently assessed before surgery,^16^ including before TKA/THA.^31^ While some investigations report that presurgical anxiety is associated with APSP,^25,28,65^ others do not replicate these conclusions.^24,26,33^ The present study provided further data on this matter by analysing bidirectional longitudinal relationships between these variables. For the global sample, APSP was predicted both by presurgical pain and presurgical anxiety levels. In fact, presurgical pain due to long-lasting osteoarthritis may underlie phenomenon of local and spreading central sensitisation with impact on pain ratings in the acute postsurgical period.^66^ Simultaneously, descending pain pathways can be modulated by emotional and cognitive processes, such as anxiety.^67^ This is line with the Gate Control and Neuromatrix theories of pain, which highlight the contribution of motivational-affective and cognitive-evaluative factors to pain experience.^68,69^ It is possible that more anxious patients are prone to increased muscle tension and to heightened attention to bodily sensations after surgery, and thus more likely to experience increased pain.^14,70^

Subsequent multigroup analyses showed differences according to the type of arthroplasty. Presurgical anxiety levels predicted APSP after TKA, but not after THA. Inversely, presurgical pain predicted APSP after THA, but not after TKA. Hence, anxiety seems to be a relevant variable only for patients undergoing TKA. Though the differences between paths were not significant among groups, the results suggest that predictors of APSP may differ according to the type of surgery, even when only joint arthroplasties are being considered. In fact, a systematic review concluded that the role of psychological factors seems to be stronger for TKA, though there were few studies on THA.^30,31^ It seems likely that different variables may predict short and long-term outcomes of different joint arthroplasties. There are significant differences in APSP intensity even among surgeries of the same anatomical site, reinforcing that risk factors should be investigated individually, instead of under larger categories (e.g. arthroplasty, abdominal).^71^ The specific variables that may be more relevant for each surgery remain uncertain, with more recent investigations coming to contrary conclusions on the weight of psychological variables for TKA and THA.^33,54^ It is now important that studies aiming to identify predictors of APSP include between-surgery comparisons. Deriving general conclusions from one surgical model may introduce relevant biases in the final conclusions.

Postsurgical anxiety was exclusively predicted by its presurgical counterpart, with no relevant influence of presurgical pain. This reinforces the utility of specific presurgical interventions targeting anxiety, with a potential beneficial effect on acute pain and recovery.^20^

The role of psychological variables was further scrutinised through mediation analyses. Despite the absence of total or direct effects, an interesting result emerged for THA when pain catastrophizing was a mediator, revealing a statistically significant indirect effect in the association between presurgical anxiety levels and APSP. Indeed, current views of mediation analysis do not require an association between independent variables and outcomes as a prerequisite to establish significant indirect effects.^47,72^ According to our data, higher presurgical anxiety was associated with higher pain catastrophizing, which predicts more intense APSP in THA. Comparable findings have been reported for hysterectomy surgery, wherein pain catastrophizing also emerged as a mediator between anxiety and APSP.^73^ The mediating role of pain catastrophizing has also been recently evidenced in osteoarthritis patients,^74,75^ further supporting the relevance of this construct for pain outcomes. Actually, pain catastrophizing has been pinpointed as the most consistent predictor of APSP,^16,31^ also with a relevant role for chronic postsurgical pain.^76^ The idea that different variables may predict APSP after TKA or THA received further support in this study. The analyses revealed that anxiety had a direct effect in pain after TKA, and an indirect effect through pain catastrophizing in pain after THA. The reasons for this difference are not straightforward but may be due to specific group characteristics at baseline. Since THA patients had higher disability and lower self-efficacy, it is plausible that these patients view their painful condition and the surgical procedure as more threatening events for which they may lack the adequate coping resources. In face of this, anxiety symptoms may trigger catastrophizing thoughts that in turn contribute to increased pain.

Despite differences between types of arthroplasties, the present results highlight the role of emotional and cognitive factors for acute pain after both procedures, namely, anxiety and pain catastrophizing. Psychological variables contribute to APSP, along with the physiological processes related to tissue injury.^77^ Thus, the biopsychosocial model should be adopted also for acute pain management.^13^ Presurgical psychological interventions can improve pain control, reduce negative mood and improve surgical recovery,^78,79^ as recognised by current guidelines for APSP control.^17^

This study has some limitations to be acknowledged. The age and sex discrepancy in patients undergoing TKA or THA is worth noting, since the first group included mostly women and had a higher mean age. The effect of these variables was controlled by including them as covariates in the CLPM models. Additionally, postsurgical pain was assessed at one time point. It is likely that an assessment with more than one evaluation moment during the acute period would provide a more comprehensive depiction of patients’ pain. Similarly, there are other psychological variables that would be relevant to consider in the context of postsurgical pain for a more comprehensive patient characterisation (i.e. fear of surgery, kinesiophobia, locus of control, resilience). These should be included in future studies to investigate their impact on APSP. Future studies should also be designed to investigate how pre/postsurgical pain and anxiety levels interact to impact longer-term outcomes. Though the longitudinal measurement invariance of anxiety was guaranteed, our decision was not to evaluate the measurement invariance of pain assessment since it was based on only two items from the BPI, which hindered this analysis.

To ensure real-world data, there was no restriction in terms of the anaesthetic and analgesic protocols.

### Conclusions

It is still unclear if and which individual characteristics weigh differently on TKA and THA outcomes. This study provided further data on this subject, by revealing different variables associated with APSP after each arthroplasty type. Presurgical anxiety levels were shown to have a direct effect in pain experience after TKA, but an indirect effect in pain after THA. For THA patients, the anxiety – pain association operated through pain catastrophizing (indirect effect): higher anxiety contributed to more catastrophizing about pain, which led to increased pain perception. Taken separately, these findings are insufficient to recommend differential psychological evaluation for TKA and THA patients but are crucial in reinforcing the need for such an assessment in both groups and the relevance of more research comparing TKA and THA. Future studies should seek to explore and specify differences between arthroplasty types and understand the reasons that may underlie them. In order to develop, test and implement effective interventions for postsurgical pain, it is crucial to identify risk factors associated with APSP after each type of surgery. Besides the humanistic reasons that warrant effective pain control at any stage, the critical contribution of APSP to developing chronic postsurgical pain should be acknowledged. Effective management of APSP may be an important measure to prevent pain chronification and reduce the associated health care costs.

## Supplemental Material

Supplemental Material - Differences in the relationship between pain and anxiety in total knee and hip arthroplasty: a longitudinal cross‐lagged analysis mediated by depression and pain catastrophizing Supplemental Material for Differences in the relationship between pain and anxiety in total knee and hip arthroplasty: a longitudinal cross‐lagged analysis mediated by depression and pain catastrophizing by Ana Cristina Paredes, Patrício Costa, Márcia Costa, Patrícia Oliveira, Pedro Varanda, Armando Almeida and Patrícia R Pinto in British Journal of Pain

## References

[1] Robinson PD McEwan J Adukia V , et al. Osteoarthritis and arthroplasty of the hip and knee. Br J Hosp Med 2018; 79: C54–C59. 10.12968/hmed.2018.79.4.C54 29620976

[2] Ringkamp M Raja SN Campbell JN , et al. Peripheral mechanisms of cutaneous nociception. In: McMahon SB Koltzenburg M Tracey I , et al. (eds). Wall and Melzack's Textbook of Pain. Philadelphia, USA: Elsevier, 2013, pp. 1–30.

[3] Gan TJ . Poorly controlled postoperative pain: prevalence, consequences, and prevention. J Pain Res 2017; 10: 2287–2298. 29026331 10.2147/JPR.S144066 PMC5626380

[4] De Boer HD . Postoperative multimodal pain management. In: Ljungqvist O Francis NK Urman RD (eds). Enhanced Recovery After Surgery (ERAS®): A Complete Guide to Optimizing Outcomes. Cham, Switzerland: Springer Nature, 2020, pp. 219–228.

[5] Deumens R Steyaert A Forget P , et al. Prevention of chronic postoperative pain: cellular, molecular, and clinical insights for mechanism-based treatment approaches. Prog Neurobiol 2013; 104: 1–37. 23410739 10.1016/j.pneurobio.2013.01.002

[6] Rawal N . Current issues in postoperative pain management. Eur J Anaesthesiol 2016; 33: 160–171. 26509324 10.1097/EJA.0000000000000366

[7] National Institute of Academic Anaesthesia (NIAA) Health Services Research Centre . Perioperative quality improvement Programme report 4 July 2021 to March 2023. London: Royal College of Anaesthetists, 2023.

[8] Borys M Zyzak K Hanych A , et al. Survey of postoperative pain control in different types of hospitals: a multicenter observational study. BMC Anesthesiol 2018; 18: 83. 30021520 10.1186/s12871-018-0551-3 PMC6052639

[9] Gerbershagen HJ Aduckathil S van Wijck AJM , et al. Pain intensity on the first day after surgery: a prospective cohort study comparing 179 surgical procedures. Anesthesiology 2013; 118: 934–944. 23392233 10.1097/ALN.0b013e31828866b3

[10] Schnabel A Yahiaoui-Doktor M Meissner W , et al. Predicting poor postoperative acute pain outcome in adults: an international, multicentre database analysis of risk factors in 50,005 patients. Pain Rep 2020; 5: e831. 32766467 10.1097/PR9.0000000000000831 PMC7390596

[11] Yang MMH Hartley RL Leung AA , et al. Preoperative predictors of poor acute postoperative pain control: a systematic review and meta-analysis. BMJ Open 2019; 9: e025091. 10.1136/bmjopen-2018-025091 PMC6500309 30940757

[12] Ip HY Abrishami A Peng PW , et al. Predictors of postoperative pain and analgesic consumption: a qualitative systematic review. Anesthesiology 2009; 111: 657–677. 19672167 10.1097/ALN.0b013e3181aae87a

[13] Small C Laycock H . Acute postoperative pain management. Br J Surg 2020; 107: e70–e80. 31903595 10.1002/bjs.11477

[14] Villemure C Bushnell MC . Cognitive modulation of pain: how do attention and emotion influence pain processing? Pain 2002; 95: 195–199. 11839418 10.1016/S0304-3959(02)00007-6

[15] Klossika I Flor H Kamping S , et al. Emotional modulation of pain: a clinical perspective. Pain 2006; 124: 264–268. 16934927 10.1016/j.pain.2006.08.007

[16] Sobol-Kwapinska M Bąbel P Plotek W , et al. Psychological correlates of acute postsurgical pain: a systematic review and meta-analysis. Eur J Pain 2016; 20: 1573–1586. 27136510 10.1002/ejp.886

[17] American Society of Anesthesiologists . Practice guidelines for acute pain management in the perioperative setting: an updated report by the American Society of Anesthesiologists Task Force on Acute Pain Management. Anesthesiology 2012; 116: 248–273. 22227789 10.1097/ALN.0b013e31823c1030

[18] Burch J Balfour A . Preoperative patient education. In: Ljungqvist O Francis NK Urman RD (eds). Enhanced Recovery After Surgery (ERAS)® - A complete guide to optimizing outcomes. Switzerland: Springer, 2020, pp. 37–49.

[19] Stamenkovic DM Rancic NK Latas MB , et al. Preoperative anxiety and implications on postoperative recovery: what can we do to change our history. Minerva Anestesiol 2018; 84: 1307–1317. 29624026 10.23736/S0375-9393.18.12520-X

[20] Gümüs K . The effects of preoperative and postoperative anxiety on the quality of recovery in patients undergoing abdominal surgery. J Perianesth Nurs 2021; 36: 174–178. 33640291 10.1016/j.jopan.2020.08.016

[21] Jellish WS O'Rourke M . Anxiolytic use in the postoperative care unit. Anesthesiol Clin 2012; 30: 467–480. 22989589 10.1016/j.anclin.2012.07.006

[22] Walburn J Vedhara K Hankins M , et al. Psychological stress and wound healing in humans: a systematic review and meta-analysis. J Psychosom Res 2009; 67: 253–271. 19686881 10.1016/j.jpsychores.2009.04.002

[23] Edwards RR Smith MT Klick B , et al. Symptoms of depression and anxiety as unique predictors of pain-related outcomes following burn injury. Ann Behav Med 2007; 34: 313–322. 18020941 10.1007/BF02874556

[24] Abrecht CR Cornelius M Wu A , et al. Prediction of pain and opioid utilization in the perioperative period in patients undergoing primary knee arthroplasty: psychophysical and psychosocial factors. Pain Med 2019; 20: 161–171. 29522115 10.1093/pm/pny020 PMC6329440

[25] Lunn TH Gaarn-Larsen L Kehlet H . Prediction of postoperative pain by preoperative pain response to heat stimulation in total knee arthroplasty. Pain 2013; 154: 1878–1885. 23769717 10.1016/j.pain.2013.06.008

[26] Luna IE Kehlet H Petersen MA , et al. Clinical, nociceptive and psychological profiling to predict acute pain after total knee arthroplasty. Acta Anaesthesiol Scand 2017; 61: 676–687. 28508511 10.1111/aas.12899

[27] Rakel BA Blodgett NP Bridget Zimmerman M , et al. Predictors of postoperative movement and resting pain following total knee replacement. Pain 2012; 153: 2192–2203. 22840570 10.1016/j.pain.2012.06.021 PMC3472094

[28] Petrovic NM Milovanovic DR Ignjatovic Ristic D , et al. Factors associated with severe postoperative pain in patients with total hip arthroplasty. Acta Orthop Traumatol Turc 2014; 48: 615–622. 25637724 10.3944/AOTT.2014.14.0177

[29] Pinto PR McIntyre T Ferrero R , et al. Predictors of acute postsurgical pain and anxiety following primary total hip and knee arthroplasty. J Pain 2013; 14: 502–515. 23541065 10.1016/j.jpain.2012.12.020

[30] Vissers MM Bussmann JB Verhaar JA , et al. Psychological factors affecting the outcome of total hip and knee arthroplasty: a systematic review. Semin Arthritis Rheum 2012; 41: 576–588. 22035624 10.1016/j.semarthrit.2011.07.003

[31] Springborg AH Visby L Kehlet H , et al. Psychological predictors of acute postoperative pain after total knee and hip arthroplasty: a systematic review. Acta Anaesthesiol Scand 2023; 67: 1322–1337. 37400963 10.1111/aas.14301

[32] Wylde V Rooker J Halliday L , et al. Acute postoperative pain at rest after hip and knee arthroplasty: severity, sensory qualities and impact on sleep. Orthop Traumatol Surg Res 2011; 97: 139–144. 21388906 10.1016/j.otsr.2010.12.003

[33] Pinto PR McIntyre T Araújo-Soares V , et al. A comparison of predictors and intensity of acute postsurgical pain in patients undergoing total hip and knee arthroplasty. J Pain Res 2017; 10: 1087–1098. 28533697 10.2147/JPR.S126467 PMC5431693

[34] Kugelman DN Mahure SA Feng JE , et al. Total knee arthroplasty is associated with greater immediate post-surgical pain and opioid use than total hip arthroplasty. Arch Orthop Trauma Surg 2022; 142: 3575–3580. 33991234 10.1007/s00402-021-03951-8

[35] Kearney MW . Cross-lagged panel analysis. In: Allen M (ed). The Sage Encyclopedia of Communication Research Methods. Thousand Oaks, California: Sage Publications, Inc, 2017, pp. 312–317.

[36] American Society of Anesthesiologists . Statement on ASA physical status classification system, 2020. https://www.asahq.org/standards-and-practice-parameters/statement-on-asa-physical-status-classification-system (accessed 22/04/2024).

[37] Cleeland C Ryan K . Pain assessment: global use of the brief pain inventory. Ann Acad Med Singapore 1994; 23: 129–138. 8080219

[38] Bellamy N Buchanan WW Goldsmith CH , et al. Validation study of WOMAC: a health status instrument for measuring clinically important patient relevant outcomes to antirheumatic drug therapy in patients with osteoarthritis of the hip or knee. J Rheumatol 1988; 15: 1833–1840. 3068365

[39] Riley JL 3rd Robinson ME . CSQ: five factors or fiction? Clin J Pain 1997; 13: 156–162. 9186023 10.1097/00002508-199706000-00010

[40] Zigmond A Snaith R . The hospital anxiety and depression scale. Acta Psychiatr Scand 1983; 67: 361–370. 6880820 10.1111/j.1600-0447.1983.tb09716.x

[41] Edwards RR Dworkin RH Turk DC , et al. Patient phenotyping in clinical trials of chronic pain treatments: IMMPACT recommendations. Pain 2016; 157: 1851–1871. 27152687 10.1097/j.pain.0000000000000602 PMC5965275

[42] Nicholas MK . The pain self-efficacy questionnaire: taking pain into account. Eur J Pain 2007; 11: 153–163. 16446108 10.1016/j.ejpain.2005.12.008

[43] Scheier MF Carver CS Bridges MW . Distinguishing optimism from neuroticism (and trait anxiety, self-mastery, and self-esteem): a reevaluation of the Life Orientation Test. J Pers Soc Psychol 1994; 67: 1063–1078. 7815302 10.1037//0022-3514.67.6.1063

[44] Diener E Emmons RA Larsen RJ , et al. The satisfaction with life scale. J Pers Assess 1985; 49: 71–75. 16367493 10.1207/s15327752jpa4901_13

[45] Melzack R . The McGill Pain Questionnaire: major properties and scoring methods. Pain 1975; 1: 277–299. 1235985 10.1016/0304-3959(75)90044-5

[46] West S Finch J Curran P . Structural equation models with nonnormal variables: problems and remedies. In: Hoyle R (ed). Structural equation modeling: Concepts, issues and applications. Thousand Oaks, CA: Sage, 1995, pp. 56–75.

[47] Hayes A . The simple mediation model. Introduction to mediation, moderation and conditional process analysis A regression-based approach. New York: The Guilford Press, 2013.

[48] Hu L Bentler PM . Cutoff criteria for fit indexes in covariance structure analysis: conventional criteria versus new alternatives. Struct Equ Model 1999; 6: 1–55.

[49] Schreiber JB Nora A Stage FK , et al. Reporting structural equation modeling and confirmatory factor analysis results: a review. J Educ Res 2006; 99: 323–338.

[50] Dabare C Le Marshall K Leung A , et al. Differences in presentation, progression and rates of arthroplasty between hip and knee osteoarthritis: observations from an osteoarthritis cohort study-a clear role for conservative management. Int J Rheum Dis 2017; 20: 1350–1360. 28493422 10.1111/1756-185X.13083 PMC5655735

[51] Hall M van der Esch M Hinman RS , et al. How does hip osteoarthritis differ from knee osteoarthritis? Osteoarthritis Cartilage 2022; 30: 32–41. 34600121 10.1016/j.joca.2021.09.010

[52] Borkhoff CM Hawker GA Wright JG . Patient gender affects the referral and recommendation for total joint arthroplasty. Clin Orthop Relat Res 2011; 469: 1829–1837. 21448775 10.1007/s11999-011-1879-x PMC3111793

[53] Katz JN Wright EA Guadagnoli E , et al. Differences between men and women undergoing major orthopedic surgery for degenerative arthritis. Arthritis Rheum 1994; 37: 687–694. 8185695 10.1002/art.1780370512

[54] Lindner M Nosseir O Keller-Pliessnig A , et al. Psychosocial predictors for outcome after total joint arthroplasty: a prospective comparison of hip and knee arthroplasty. BMC Musculoskelet Disord 2018; 19: 159. 29788969 10.1186/s12891-018-2058-y PMC5964720

[55] Roos EM Grønne DT Thorlund JB , et al. Knee and hip osteoarthritis are more alike than different in baseline characteristics and outcomes: a longitudinal study of 32,599 patients participating in supervised education and exercise therapy. Osteoarthritis Cartilage 2022; 30: 681–688. 35176479 10.1016/j.joca.2022.02.001

[56] Buis N Esfandiari H Hoch A , et al. Overview of methods to quantify invasiveness of surgical approaches in orthopedic surgery-A scoping review. Front Surg 2021; 8: 771275. 35155547 10.3389/fsurg.2021.771275 PMC8825480

[57] Helminen EE Arokoski JP Selander TA , et al. Multiple psychological factors predict pain and disability among community-dwelling knee osteoarthritis patients: a five-year prospective study. Clin Rehabil 2020; 34: 404–415. 31965830 10.1177/0269215519900533

[58] Somers TJ Keefe FJ Pells JJ , et al. Pain catastrophizing and pain-related fear in osteoarthritis patients: relationships to pain and disability. J Pain Symptom Manag 2009; 37: 863–872. 10.1016/j.jpainsymman.2008.05.009 PMC2702756 19041218

[59] López-Bravo MD Zamarrón-Cassinello MD Touche R , et al. Psychological factors associated with functional disability in patients with hip and knee osteoarthritis. Behav Med 2021; 47: 285–295. 32910744 10.1080/08964289.2020.1813682

[60] Hanssen MM Peters ML Boselie JJ , et al. Can positive affect attenuate (persistent) pain? State of the art and clinical implications. Curr Rheumatol Rep 2017; 19: 80. 29119260 10.1007/s11926-017-0703-3 PMC5683052

[61] Finan PH Garland EL . The role of positive affect in pain and its treatment. Clin J Pain 2015; 31: 177–187. 24751543 10.1097/AJP.0000000000000092 PMC4201897

[62] Cohen SP Vase L Hooten WM . Chronic pain: an update on burden, best practices, and new advances. Lancet 2021; 397: 2082–2097. 34062143 10.1016/S0140-6736(21)00393-7

[63] Basten-Günther J Peters M Lautenbacher S . Optimism and the experience of pain: a systematic review. Behav Med 2019; 45: 323–339. 30570408 10.1080/08964289.2018.1517242

[64] Niederstrasser NG Cook S . Investigating the true effect of psychological variables measured prior to arthroplastic surgery on postsurgical outcomes: a p-curve analysis. J Pain 2021; 22: 400–414. 33098977 10.1016/j.jpain.2020.07.005

[65] Thomazeau J Rouquette A Martinez V , et al. Acute pain factors predictive of post-operative pain and opioid requirement in multimodal analgesia following knee replacement. Eur J Pain 2016; 20: 822–832. 26517014 10.1002/ejp.808

[66] Arendt-Nielsen L Nie H Laursen MB , et al. Sensitization in patients with painful knee osteoarthritis. Pain 2010; 149: 573–581. 20418016 10.1016/j.pain.2010.04.003

[67] Baert IA Lluch E Mulder T , et al. Does pre-surgical central modulation of pain influence outcome after total knee replacement? A systematic review. Osteoarthritis Cartilage 2016; 24: 213–223. 26382109 10.1016/j.joca.2015.09.002

[68] Melzack R . Pain and the neuromatrix in the brain. J Dent Educ 2001; 65: 1378–1382. 11780656

[69] Melzack R Wall PD . Pain mechanisms: a new theory. Science 1965; 150: 971–979. 5320816 10.1126/science.150.3699.971

[70] Lucchetti G Oliveira AB Mercante JPP , et al. Anxiety and fear-avoidance in musculoskeletal pain. Curr Pain Headache Rep 2012; 16: 399–406. 22791352 10.1007/s11916-012-0286-7

[71] Sommer M Geurts JW Stessel B , et al. Prevalence and predictors of postoperative pain after ear, nose, and throat surgery. Arch Otolaryngol Head Neck Surg 2009; 135: 124–130. 19221238 10.1001/archoto.2009.3

[72] Rucker DD Preacher KJ Tormala ZL , et al. Mediation analysis in social psychology: current practices and new recommendations. Soc Pers Psychol Compass 2011; 5: 359–371.

[73] Pinto PR McIntyre T Almeida A , et al. The mediating role of pain catastrophizing in the relationship between presurgical anxiety and acute postsurgical pain after hysterectomy. Pain 2012; 153: 218–226. 22115922 10.1016/j.pain.2011.10.020

[74] Tighe CA Youk A Ibrahim SA , et al. Pain catastrophizing and arthritis self-efficacy as mediators of sleep disturbance and osteoarthritis symptom severity. Pain Med 2020; 21: 501–510. 31504838 10.1093/pm/pnz187 PMC8483157

[75] Rausa M Spada GE Patron E , et al. Do catastrophizing and autonomic-reduced flexibility mediate pain outcomes in chronic headache? Neurol Sci 2022; 43: 3283–3295. 34799749 10.1007/s10072-021-05732-y

[76] Theunissen M Peters ML Bruce J , et al. Preoperative anxiety and catastrophizing: a systematic review and meta-analysis of the association with chronic postsurgical pain. Clin J Pain 2012; 28: 819–841. 22760489 10.1097/AJP.0b013e31824549d6

[77] Buvanendran A Lubenow TR Kroin JS . Postoperative pain and its management. In: McMahon SB Koltzenburg M Tracey I , et al. (eds). Wall and Melzack's Textbook of Pain. Philadelphia, USA: Elsevier, 2013, pp. 629–644.

[78] Powell R Scott NW Manyande A , et al. Psychological preparation and postoperative outcomes for adults undergoing surgery under general anaesthesia. Cochrane Database Syst Rev 2016; 2016: Cd008646. 27228096 10.1002/14651858.CD008646.pub2 PMC8687603

[79] Tong F Dannaway J Enke O , et al. Effect of preoperative psychological interventions on elective orthopaedic surgery outcomes: a systematic review and meta-analysis. ANZ J Surg 2020; 90: 230–236. 31334592 10.1111/ans.15332

