# Supplementary material for: Differences in the relationship between pain and anxiety in total knee and hip arthroplasty: a longitudinal cross‐lagged analysis mediated by depression and pain catastrophizing
Source: Br J Pain. 2024 Aug 17;18(6):482–96. doi: 10.1177/20494637241273905 (PMC11561940; doi:10.1177/20494637241273905)

Electronic Supplementary Material 1

Surgical and anesthetic procedures

Most TKA were performed through medial parapatellar approach (107, 89.1%) and most THA were performed via posterior approach (99, 90.8%). Anesthetic and analgesic procedures were determined individually by the anesthesiologist in charge, according to established protocols, medical necessity, or patient preference. Anesthesia was either locoregional only (TKA: 116, 96.7%; THA: 103, 94.5%) or locoregional plus general anesthesia (TKA: 4, 3.3%; THA: 6, 5.5%). Locoregional anesthesia was subarachnoid block (TKA: 114, 95%; THA: 103, 94.5%) or epidural block (TKA: 2, 1.7%, THA: 0). These were achieved with a combination of bupivacaine (0.5%/10ml) or hyperbaric bupivacaine (20mg/4ml) and sufentanil (0.005mg/ml). Peripheral nerve block was achieved with rocuronium bromide (10mg/ml) in conjunction with perineural ropivacaine (7.5mg/ml) or lidocaine (10mg/ml).

Postsurgical analgesia was administered by epidural, perineural or intravenous routes. Coadjuvant analgesics (detailed below) were delivered via oral or intravenous route as prescribed, with an indication to administer rescue analgesia if pain intensity was 3 or higher on a 0-10 numerical rating scale (NRS). Epidural analgesia was administered either by programmed intermittent epidural bolus (PIEB), (TKA: 4, 3.3%; THA: 0), by continuous disposable infusion balloon (DIB, TKA: 78, 65%; THA: 5, 4.6%) or by patient-controlled epidural analgesia (PCEA) (TKA: 17, 14.2%; THA: 7, 6.4%) with background infusion. These protocols included a combination of ropivacaine (0.15%) and fentanyl (1.5µg/ml) and other analgesics such as paracetamol (1g, 6/6h) and non-steroidal anti-inflammatory drugs (NSAIDs: ketorolac 30mg, parecoxib 40mg or diclofenac 50mg,

12/12h). Rescue analgesia was prescribed individually and administered as needed (ropivacaine 40mg/20ml, tramadol 100mg, metamizole 2g, pethidine 25 mg). Perineural analgesia (TKA: 2, 2.5%; THA: 0) was delivered through patient-controlled balloon pump (patient-controlled regional analgesia, PCRA) with ropivacaine (1.5mg/ml). Coadjuvant medication was paracetamol (1g) and tramadol (100mg).

Intravenous analgesia (TKA: 6, 5%; THA: 96, 88.1%) consisted of different combinations of paracetamol (1g, 6/6h), ketorolac (30 mg, 12/12h) and tramadol (100mg, 8/8h) and additional medication (pethidine, metamizole) delivered in case of uncontrolled pain (NRS ≥ 3). Some patients had an intravenous DIB protocol (TKA: 12, 10%; THA: 1, 0.9%), with a 5ml/hour drip of tramadol (400 mg) and droperidol (5mg), along with coadjuvant medication (paracetamol and NSAIDSs) and rescue analgesia (pethidine, 20 mg).

Antiemetic treatment (metoclopramide or ondansetron) was used as needed and all patients underwent infection prophylaxis with antibiotics (cefazolin) and thromboembolism prophylaxis with LMWH (low molecular weight heparin – enoxaparin).

For both types of arthroplasties, the success of surgery was evaluated through control x-rays taken after discharge from the post-anesthesia care unit to confirm prosthesis placement and alignment and monitor early complications. Walking training was initiated one day after surgery. At discharge from the hospital, achieving 0-90 degrees of flexion at the operated joint was a further indicator of success.

Supplementary Material 2

Supplementary figure 1. Representation of the model for assessing longitudinal

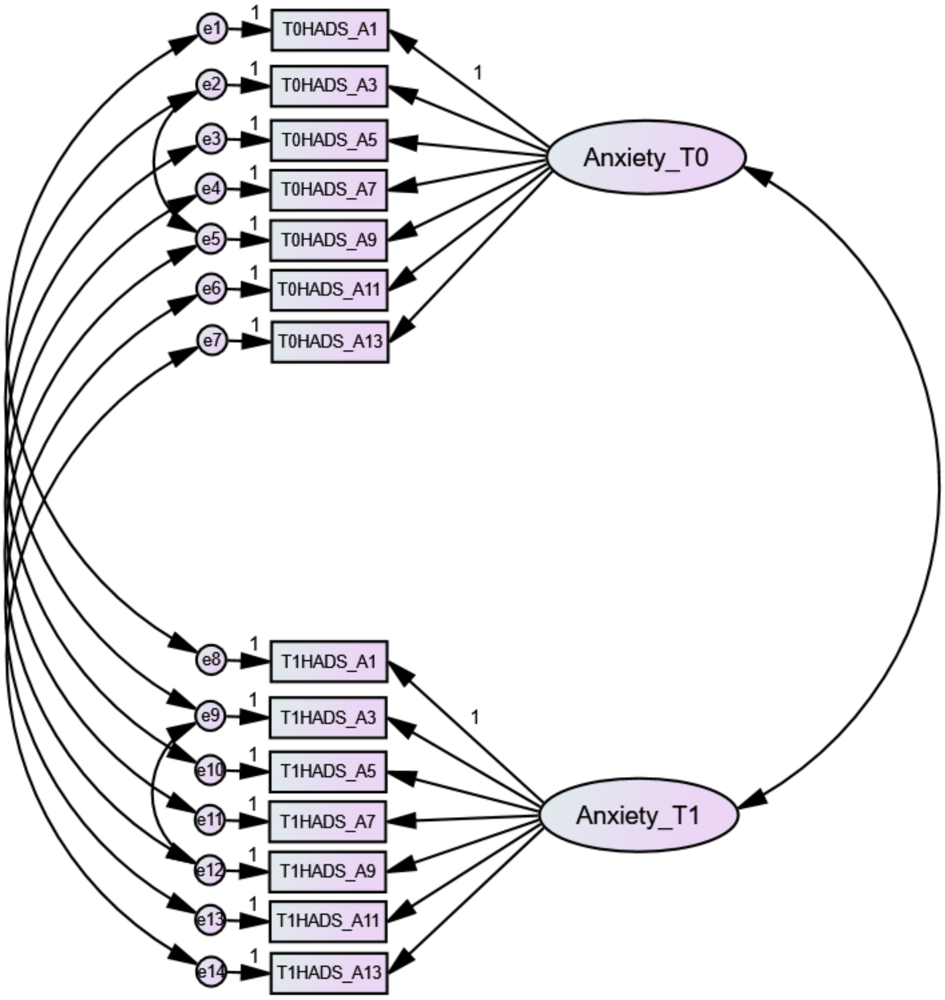

measurement invariance.

Supplementary Material 3

Supplementary figure 2. Cross-lagged panel models (no mediator) with standard estimates for the global sample, TKA and THA.

GLOBAL SAMPLE

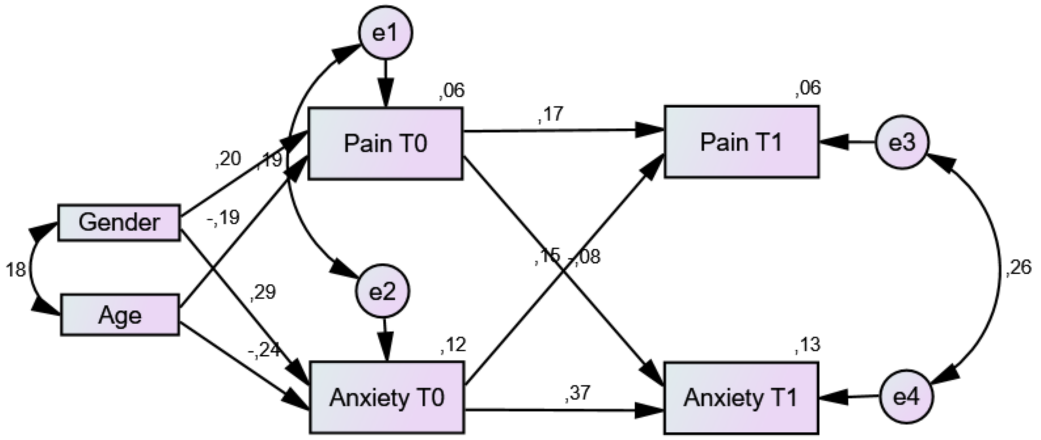

TOTAL KNEE ARTHROPLASTY

TOTAL HIP ARTHROPLASTY

Supplementary Material 4

Supplementary figure 3. Cross-lagged panel models with potential mediator variables (depression and pain catastrophizing) and standard estimates for the global sample, TKA and

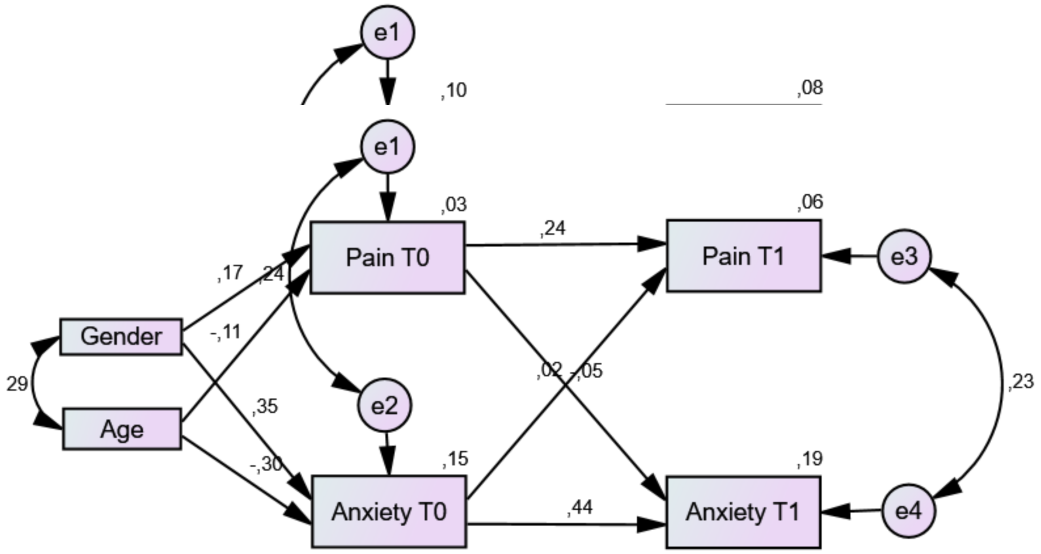

THA.

<u>Mediator: depression</u>

GLOBAL SAMPLE

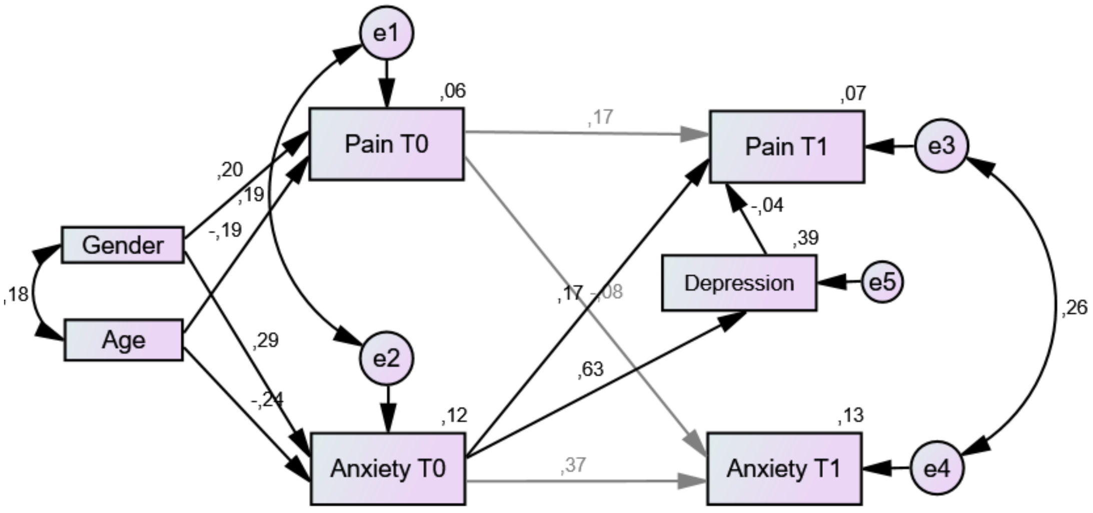

TOTAL KNEE ARTHROPLASTY

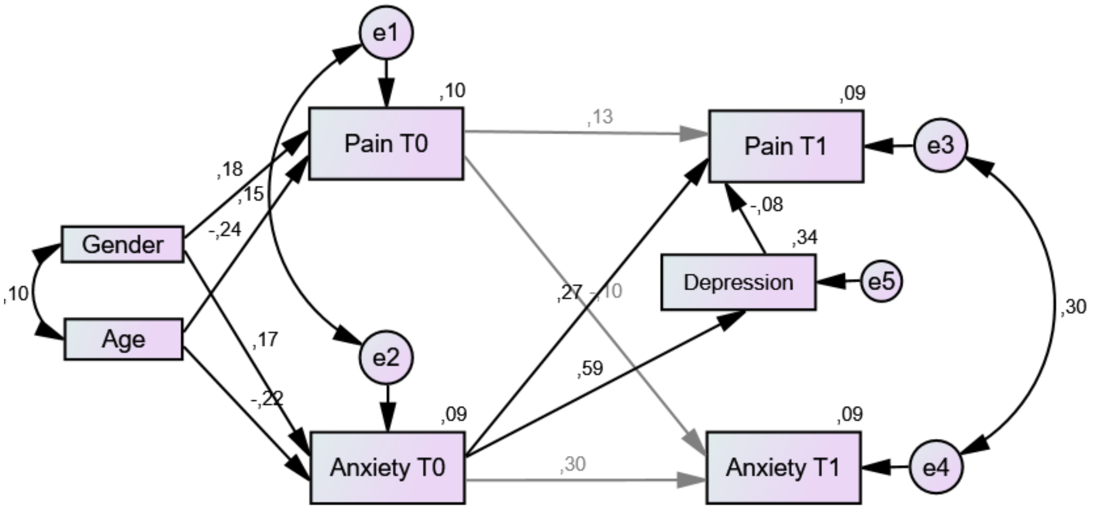

TOTAL HIP ARTHROPLASTY

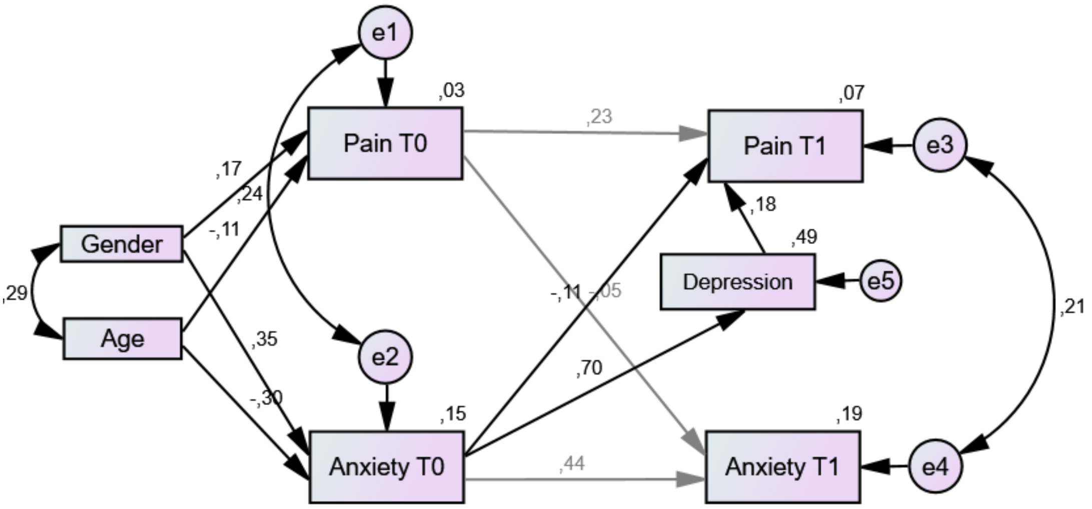

<u>Mediator: pain catastrophizing</u>

GLOBAL SAMPLE

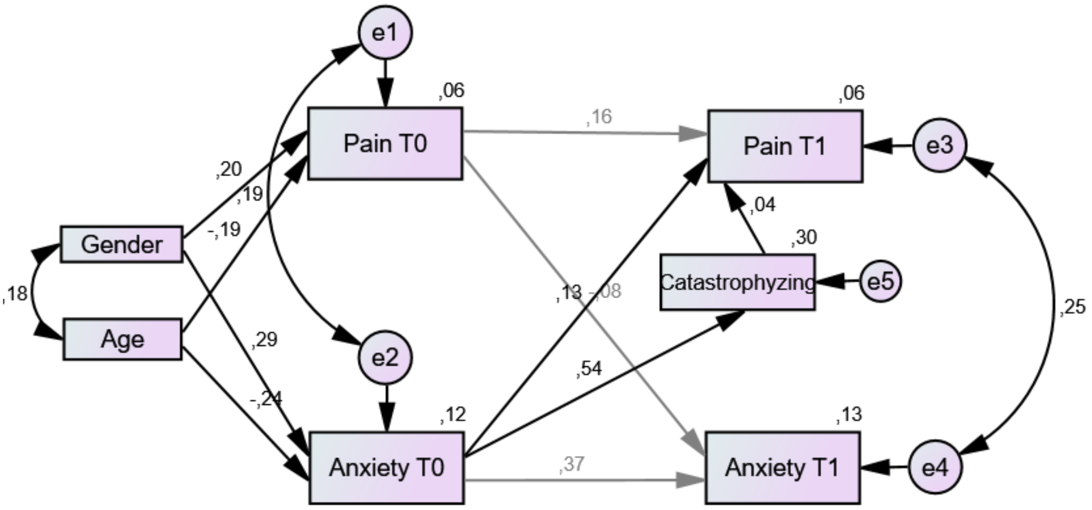

TOTAL KNEE ARTHROPLASTY

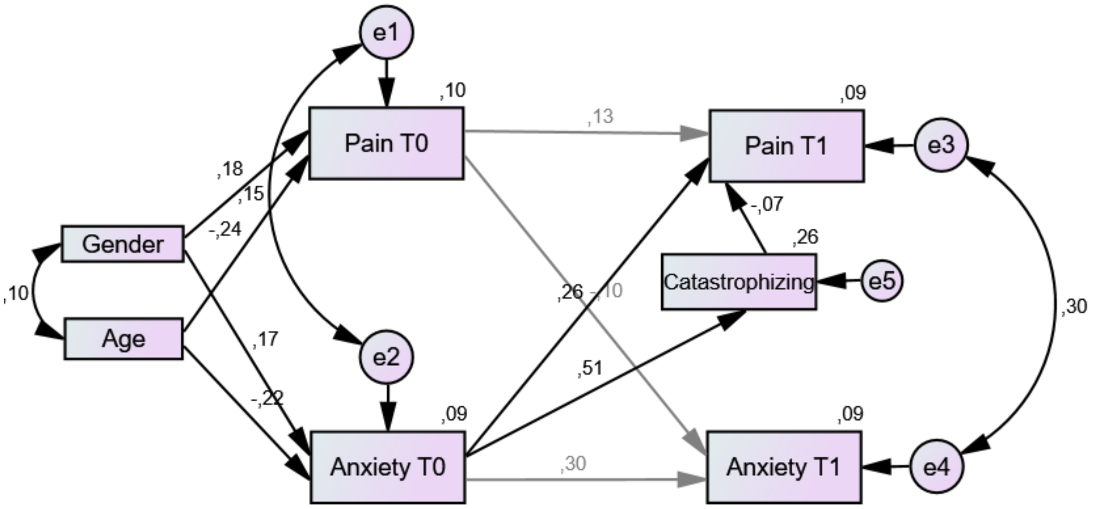

TOTAL HIP ARTHROPLASTY

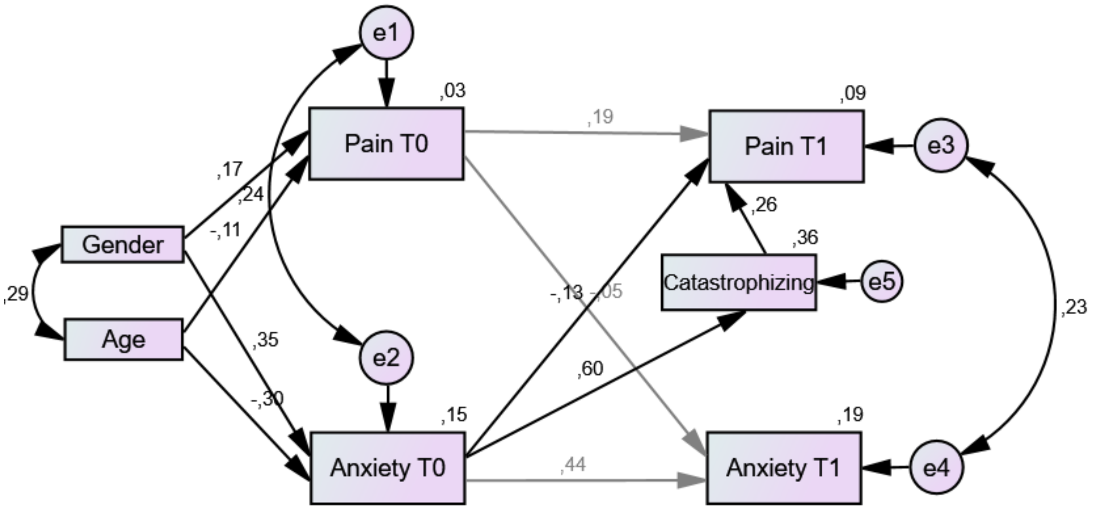

Supplement: Supplemental Material - Differences in the relationship between pain and anxiety in total knee and hip arthroplasty: a longitudinal cross‐lagged analysis mediated by depression and pain catastrophizing [file sj-pdf-1-bjp-10.1177_20494637241273905.pdf]
